# Development and evaluation of a multiplex PCR for simultaneous detection of *Mycobacterium tuberculosis* complex, *Mycobacterium avium* complex, and *Burkholderia pseudomallei* in clinical specimens

**DOI:** 10.1371/journal.pntd.0014730

**Published:** 2026-09-24

**Authors:** Hasyanee Binmaeil, Thanakon Bunsong, Janjira Thaipadungpanit, Wipawee Saenwongsa, Montira Ngoenpramual, Sutthinan Chawsorngkorn, Soawarat Deekae, Sunee Chayangsu, Wang Nguitragool, Narisara Chantratita

**Affiliations:** 1 Department of Microbiology and Immunology, Faculty of Tropical Medicine, Mahidol University, Bangkok, Thailand; 2 Department of Clinical Tropical Medicine, Faculty of Tropical Medicine, Mahidol University, Bangkok, Thailand; 3 Mahidol-Oxford Tropical Medicine Research Unit, Faculty of Tropical Medicine, Mahidol University, Bangkok, Thailand; 4 Office of Disease Prevention and Control Region 10^th^, Ministry of Public Health, Ubon Ratchathani, Thailand; 5 Department of Medical Technology and Clinical Pathology, Surin Hospital, Surin, Thailand; 6 Department of Internal Medicine, Surin Hospital, Surin, Thailand; 7 Department of Molecular Tropical Medicine and Genetics, Faculty of Tropical Medicine, Mahidol University, Bangkok, Thailand; 8 The Academy of Science, The Royal Society of Thailand, Bangkok, Thailand; Tufts Medical Center, UNITED STATES OF AMERICA

## Abstract

Effective control of tuberculosis, nontuberculous mycobacterial (NTM) disease, and melioidosis depends on rapid and accurate laboratory diagnosis. *Mycobacterium tuberculosis* complex (MTBC) remains a health priority, whereas NTM infections, particularly *Mycobacterium avium* complex (MAC), are increasingly recognized and may be misdiagnosed as tuberculosis. Melioidosis, caused by *Burkholderia pseudomallei*, is underdiagnosed in endemic regions. These infections have overlapping clinical presentations, and conventional diagnostics methods may not accurately differentiate among these diseases. We developed and evaluated a quadruplex real-time PCR assay (TAM-mPCR) for simultaneous detection of MTBC, MAC, and *B. pseudomallei*, targeting *IS6110*, *rrs*, and *BPSS1187*. A total of 607 pulmonary and extrapulmonary specimens from patients with suspected tuberculosis were collected at two sites in Thailand between 2024 and 2025. The limits of detection were 2 pg/reaction for MTBC, 0.1 ng/reaction for MAC, and 50 fg/reaction for *B. pseudomallei*, with 100% analytical specificity. For MTBC detection, TAM-mPCR demonstrated 100% sensitivity (330/330) and 99% (189/191) specificity. All three culture-confirmed MAC specimens were detected. For *B. pseudomallei*, the assay demonstrated 73.2% sensitivity (30/41) and 100% specificity (79/79). For MTBC, TAM-mPCR showed high concordance with NAAT and culture results, but differed significantly from AFB microscopy (*P* < 0.001). The assay also detected additional MTBC, MAC, and *B. pseudomallei* infections that were missed by routine diagnostics. TAM-mPCR enables rapid simultaneous pathogen detection and differentiation and may improve case detection and patient management in setting where tuberculosis and melioidosis co-endemic.

## Author summary

Accurate diagnosis of tuberculosis, nontuberculous mycobacterial (NTM) disease, and melioidosis is challenging due to overlapping clinical presentations and limitations of routine diagnostic methods. We developed a multiplex real-time PCR assay (TAM-mPCR; Tuberculosis–Avium–Melioidosis-mPCR) for simultaneous detection of *Mycobacterium tuberculosis* complex (MTBC)*, Mycobacterium avium* complex (MAC)*,* and *B. pseudomallei* directly from clinical specimens. The assay was evaluated using 607 pulmonary and extrapulmonary specimens from patients with suspected tuberculosis in northeastern Thailand. The assay demonstrated high sensitivity and specificity across specimen types and showed strong concordance with nucleic acid amplification tests (NAAT) and bacterial culture. TAM-mPCR detected all three culture-confirmed MAC specimens and showed moderate sensitivity and 100% specificity for *B. pseudomallei*. Importantly, the assay identified additional MTBC, MAC, and *B. pseudomallei* infections, including MTBC–*B. pseudomallei* co-infections, among specimens with negative routine diagnostic results. These finding highlight the potential of TAM-mPCR to improve detection of clinically significant infections and support more accurate diagnosis in co-endemic settings.

## Introduction

Tuberculosis (TB) and melioidosis are major infectious diseases of considerable public health importance across tropical regions. Both diseases are associated with high morbidity and mortality, particularly in Southeast Asia, where environmental exposures, socioeconomic factors, and limited access to healthcare contribute to a substantial disease burden. In Thailand, more than 2,500 culture-confirmed melioidosis cases are reported annually, with an estimated case fatality rate of approximately 40% [1–3], while TB remains one of the leading global causes of infectious diseases worldwide [4]. The two diseases frequently present with overlapping clinical manifestations, including prolonged fever, pneumonia, and abscess formation, and share common risk factors such as diabetes mellitus and immunosuppression [5,6]. These similarities often lead to diagnostic uncertainty, treatment delays, and inappropriate clinical management.

Melioidosis, caused by *Burkholderia pseudomallei*, is a major cause of community-acquired sepsis in endemic areas. This environmental saprophyte, commonly found in soil and surface water, infects humans through inoculation, inhalation, or ingestion. The most common clinical presentation of melioidosis is lung infection, occurring in approximately half of all cases [7]. Globally, an estimated 165,000 cases and 89,000 deaths occur annually, with the highest burden reported in Southeast Asia and northern Australia [1]. TB can be classified based on the site of disease into pulmonary tuberculosis (PTB) or extrapulmonary tuberculosis (EPTB). PTB, caused by the *Mycobacterium tuberculosis* complex (MTBC), remains the most common form of the disease and accounted for an estimated 10.8 million new cases and 1.25 million deaths globally in 2023, with Southeast Asia contributing approximately 45% of the total burden [4]. In contrast, EPTB refer to TB occurring outside the lungs, represents approximately 15–20% of all TB cases. It can occur in almost any part of the body, most commonly the lymph nodes, pleura, and bones and joints [8]. Although effective treatments exist for drug-sensitive TB, the prolonged six-month treatment regimen poses significant challenges for patients and health systems. Moreover, the emergence of multidrug-resistant (MDR) and extensively drug-resistant (XDR) TB further exacerbates clinical and public health concerns [9,10]. Conventional culture-based diagnostics, although considered the gold standard, require prolonged incubation and specialized biosafety infrastructure and have low sensitivity in paucibacillary infections, resulting in delayed case detection and ongoing transmission [11,12]. The WHO recommends Xpert MTB/RIF or Xpert Ultra as initial tests for tuberculosis and rifampicin resistance in individuals with presumptive tuberculosis [13]. In Thailand, Xpert MTB/RIF is used in routine clinical practice and is primarily applied among high-risk patients, particularly those suspected of MDR-TB [14].

Nontuberculous mycobacteria (NTM) are increasingly recognized as important opportunistic pathogens causing chronic pulmonary and extrapulmonary diseases that resemble TB in both clinical presentation and radiographic appearance [15,16]. NTM comprise all *Mycobacterium* species other than MTBC and *M. leprae* [17] and are widely distributed in soil and water [18,19]. More than 190 species and subspecies have been described, with the *M. avium* complex (MAC), *M. abscessus*, and *M. kansasii* being most frequently associated with human disease [20]. MAC is the most common cause of NTM disease in TB-endemic settings, particularly in high-burden regions such as Southeast Asia, the Western Pacific, and Africa, and frequently mimics tuberculosis in clinical practice, leading to diagnostic challenges [21–23]. Although the incidence of NTM disease has increased in several Asian countries, particularly Japan, South Korea, and Taiwan, its epidemiology in many tropical settings remains poorly characterized [24–26]. Because pulmonary NTM disease can resemble tuberculosis clinically and radiographically, failure to identify the causative species may lead to inappropriate treatment and underestimation of the disease burden [15,16].

Co-infection cases of *M. tuberculosis* and *B. pseudomallei* have been reported over the past decade, particularly in India and the WHO Western Pacific Region [27]. These cases are associated with severe symptoms and high mortality. Patients with symptoms suspected of MTBC, NTM or *B. pseudomallei* infections are common tropical countries, highlighting the importance of considering these pathogens in the differential diagnosis. Melioidosis can closely mimic TB clinically and radiographically. Patients are often treated for one infection alone, and co-infections may therefore only be recognized once culture results are confirmed by culture positive for both pathogens [5,6,28]. The shared epidemiological risk groups, including individuals with diabetes mellitus, HIV infection, and chronic kidney disease, likely contribute to under-recognition melioidosis cases with undifferentiated symptoms of PTB [5,6,28].

Current diagnostic assays used in routine hospital settings are unable to simultaneously and accurately detect MTBC, NTM and melioidosis in patients presenting with TB–like symptoms, particularly in resource-limited settings. Although culture remains the gold standard, it is time-consuming, resource-intensive, and largely restricted to regional or provincial laboratories [5,6,29]. AFB staining is a rapid and low-cost screening tool for *Mycobacterium* spp.; however, it has limited sensitivity and lacks species-level discrimination [29]. Molecular methods, including singleplex and multiplex PCR assays as well as the commercial GeneXpert MTB/RIF, have demonstrated superior sensitivity and specificity compared to conventional approaches [29–35]. Nevertheless, most PCR-based assays target a single pathogen, requiring additional time, labor, and cost to determine the etiology of pulmonary infections.

Previous multiplex PCR panels have enabled the simultaneous detection and differentiation of MTBC, MAC, and other clinically relevant mycobacteria using 16S rRNA, and other mycobacterial targets [36–38]. However, these panels were designed primarily for mycobacterial identification and did not include *B. pseudomallei*. Therefore, separate diagnostic assays are generally required to distinguish mycobacterial infections from melioidosis in patients with overlapping clinical presentations.

Tropical regions are co-endemic for both tuberculosis and melioidosis, underscoring the need for a more comprehensive diagnostic approach. To address this gap, we developed an in-house quadruplex real-time PCR assay, termed TAM-mPCR, for the simultaneous detection of MTBC, MAC, and *B. pseudomallei* in a single reaction. *IS6110* was selected as an MTBC-specific target because of its established use for the molecular detection of MTBC [39]. The *rrs* target was selected to detect a conserved 16S rRNA region in MTBC and MAC and, in combination with *IS6110*, to support differentiation of their amplification patterns [38,40]. *BPSS1187* was selected as a *B. pseudomallei*-specific target based on its previously demonstrated analytical specificity [41,42]. Human *RNase P* was included as an internal control for specimen adequacy, DNA extraction, and PCR amplification [43]. The assay was evaluated using 607 clinical specimens collected from two sites (the Office of Disease Prevention and Control Region 10^th^, Ubon Ratchathani and Surin Hospital) representing hospitals across six provinces in Northeast Thailand. This study assesses its diagnostic performance of TAM-mPCR and explores its potential to improve detection accuracy and identify underrecognized tropical diseases and co-infections in co-endemic settings.

## Materials and Methods

### Ethical approval

This study was approved by the Ethics Committee of the Faculty of Tropical Medicine, Mahidol University (MUTM 2024-069-01), and Surin hospital (100/2567). Written informed consent was not obtained from participants because only de-identified residual clinical specimens collected for routine diagnostic purposes were used, and no additional procedures or patient information were required.

### Clinical specimens and DNA extraction

**Fig 1:**
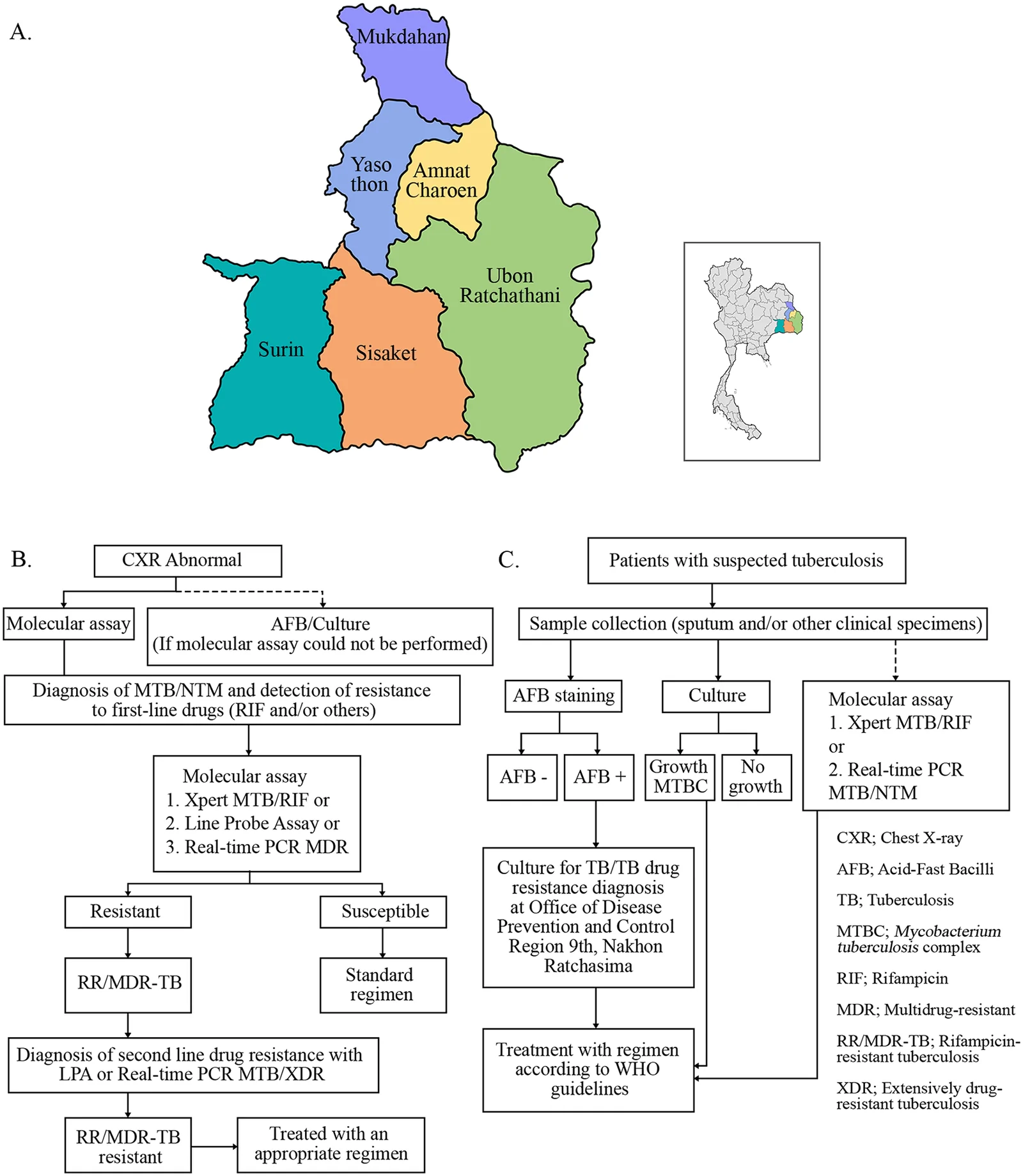
Study area showing the six provinces where clinical specimens were collected in this study. Ubon Ratchathani serves as a regional reference laboratory covering five provinces in northeastern Thailand: Ubon Ratchathani, Sisaket, Yasothon, Amnat Charoen, and Mukdahan. **(A)**. Flowchart of mycobacteria detection and antibiotic susceptibility testing at the Office of Disease Prevention and Control Region 10^th^, Ubon Ratchathani **(B)**, and Surin Hospital **(C)**. Patients with clinical suspicion of pulmonary TB or abnormal chest radiography underwent acid-fast bacilli (AFB) staining, molecular assays, and/or culture. Drug-susceptible tuberculosis cases were treated with the standard first-line anti-tuberculosis regimen, particularly rifampicin and/or isoniazid. Patients were classified as drug-susceptible TB or rifampicin-resistant/multidrug-resistant TB (RR/MDR-TB), and appropriate treatment was initiated. For patients with drug resistance, second-line resistance testing was performed using line probe assays or advanced molecular methods (e.g., real-time PCR assays for XDR-TB) to guide treatment. CXR, chest X-ray; AFB, acid-fast bacilli; TB, tuberculosis; MTBC, *Mycobacterium tuberculosis* complex; NTM, nontuberculous mycobacteria; LPA, line probe assay; RIF, rifampicin; MDR, multidrug-resistant; RR/MDR-TB, rifampicin-resistant or multidrug-resistant tuberculosis; XDR, extensively drug-resistant tuberculosis; WHO, World Health Organization.

A total of 607 anonymized clinical specimens were collected from two study sites: 420 clinical specimens collected from the Office of Disease Prevention and Control Region 10^th^, Ubon Ratchathani between 25 September 2024 and 31 August 2025, and 187 specimens collected from Surin Hospital, between 30 October 2024 and 31 August 2025 (Fig 1A). All samples were leftover clinical specimens or DNA samples that had undergone routine diagnostic testing at the respective study sites. Among these, 504 (83.0%) were pulmonary specimens and 103 (17.0%) were extrapulmonary specimens (Table 1).

**Table 1:** Sources and clinical specimen types.

Specimens	Ubon Ratchathani Center	Surin Hospital	Total
**Pulmonary**	**351**	**153**	**504**
Sputum	351	147	498
Bronchoalveolar lavage	0	6	6
**Extrapulmonary**	**69**	**34**	**103**
Pleural fluid (pleural effusion)	21	8	29
Cerebrospinal fluid	19	3	22
Tissue	12	0	12
Pus	5	10	15
Peritoneal fluid/ascitic fluid	5	1	6
Stool	2	0	2
Pericardial fluid	1	7	8
Spinal tissue	1	0	1
Lymph node (supraclavicular)	1	0	1
Synovial fluid (knee fluid)	0	4	4
Urine	0	1	1
Other body fluids (unspecified)	2	0	2
Total	420	187	607

Prior to DNA extraction, specimens were collected and subjected to pre-treatment. Pus was collected using sterile cell harvesters and resuspended in 500 µL of phosphate-buffered saline (PBS). Sputum was decontaminated differently between study sites. At Ubon Ratchathani center, sputum was treated with an equal volume of 2% N-acetyl-L-cysteine (NALC)/NaOH solution, whereas at Surin hospital, sputum was treated with an equal volume of 4% NaOH.

DNA extraction was then performed according to routine laboratory protocols at each site. At Ubon Ratchathani center, DNA was extracted using the magLEAD 12gC system (Precision System Science Co., Ltd., Chiba, Japan). Briefly, 200 µL of pretreated specimens were subjected to nucleic acid extraction using a MagDEA Dx SV reagent. The procedure included sample lysis, proteinase K digestion, binding of nucleic acids to magnetic beads, washing, and elution steps. A total of 50 µL of purified nucleic acid was obtained, of which 5–10 µL was used for routine molecular testing. The remaining DNA extract was stored at −20 °C until use. At Surin Hospital, clinical specimens were stored at −80 °C prior to DNA extraction. DNA was extracted from samples using the QIAamp DNA Mini Kit (Qiagen, Hilden, Germany), with a final elution volume of 70 µL. Purified DNA was stored at −20 °C until use. The sources and types of clinical specimens are summarized in Table 1.

### TB cases identified at study sites

TB diagnosis at both Ubon Ratchathani center and Surin hospital was performed in accordance with the Thailand National Tuberculosis Control Guidelines (2021), aligned with recommendations from the World Health Organization (WHO) guidelines for TB diagnosis and drug resistance detection (2021 and 2023).

The Office of Disease Prevention and Control Region 10^th^, Ubon Ratchathani, Thailand serves as a regional reference center covering five provinces in northeastern Thailand: Ubon Ratchathani, Sisaket, Yasothon, Amnat Charoen, and Mukdahan (Fig 1A).

At the Ubon Ratchathani Center, individuals with clinical suspicion of TB or abnormal chest radiography underwent initial testing using molecular diagnostic assays as first-line diagnostic tools, including Xpert MTB/RIF Ultra (Cepheid, USA), line probe assay (GenoType MTBDRplus v2.0; Hain Lifescience, Germany), and/or real-time PCR assays for detection of MTBC and drug resistance (e.g., cobas MTB-RIF/INH, Roche Molecular Systems, USA; or Anyplex MTB/NTM Real-Time Detection Assay v2.0, Seegene Inc., Republic of Korea). Based on the molecular results, patients were classified as drug-susceptible TB or rifampicin-resistant/multidrug-resistant TB (RR/MDR-TB), and appropriate treatment was initiated. When molecular testing could not be performed due to reagent unavailability, AFB smear and mycobacterial culture were used as alternative diagnostic approaches. If patients were MDR-TB on first-line drugs, second-line drug susceptibility testing was performed using line probe assays or advanced molecular methods (e.g., real-time PCR for XDR-TB) to guide treatment (Fig 1B).

At Surin Hospital, clinical specimens of patients with suspected tuberculosis, with or without abnormal chest radiography findings, were subjected to AFB staining and/or cultured on Ogawa medium. Specimens were subsequently referred to the Disease Prevention and Region 9^th^, Nakhon Ratchasima, Thailand for culture confirmation of MTBC, molecular diagnostic testing and drug susceptibility testing. Molecular assays, such as Xpert MTB/RIF or real-time PCR for MTB/NTM, were performed at Surin Hospital as clinically indicated (Fig 1C).

### Reference standards for the identification of MTBC, MAC, and *B. pseudomallei*

Reference standards were defined separately for each target and used to evaluate the diagnostic performance of the TAM-mPCR assay. A specimen was classified as MTBC-positive if MTBC was detected by at least one routine nucleic acid amplification test (NAAT) performed at the study site, including real-time PCR, Xpert MTB/RIF, or a line probe assay (LPA), and/or if MTBC was isolated by mycobacterial culture. AFB smear-positive specimens without a positive NAAT or mycobacterial culture were excluded from the diagnostic-performance analysis. A specimen was classified as MAC-positive when mycobacterial culture yielded MAC growth with species confirmation. A specimen was classified as *B. pseudomallei*-positive when *B. pseudomallei* was isolated by bacterial culture at the study site. Co-infection with MTBC and *B. pseudomallei* was defined as evidence of MTBC by NAAT and/or mycobacterial culture together with evidence of *B. pseudomallei* by bacterial culture or sequencing. Samples not tested by either the reference standard or TAM-mPCR were excluded from the analysis.

### Development of Quadruplex real-time PCR assay for tuberculosis and melioidosis

An in-house quadruplex real-time PCR assay, termed TAM-mPCR was developed using primers and probes targeting (i) the MTBC-specific insertion sequence *IS6110*, (ii) the conserved 16S ribosomal RNA region of MTBC and MAC (*rrs*), (iii) *BPSS1187* of *B. pseudomallei* [41,42], and (iv) the human *RNase P* gene, which served as an internal control for DNA extraction and PCR assay controls [43] (S1 Table).

The TAM-mPCR assay was performed in a total volume of 20 µL containing a 4 × Multiplex PCR Master Mix (QuantiNova Multiplex PCR Kit, Qiagen, Australia), 0.8 µM *RNase P* primers, 0.4 µM *IS6110* primer, 0.4 µM *rrs* primer and *BPSS1187* primer, 0.25 µM of each TaqMan probe, 2.5 µL template DNA. The reaction was performed using CFX96 Touch Real-Time PCR System (Bio-Rad, Hercules, CA, USA) with an initial denaturation at 95°C for 2 min, followed by 45 cycles of denaturation at 95°C for 5 seconds and combined annealing/extension step at 60°C for 30 seconds. Positive and negative (no-template) controls were included in each screening. Each fluorescence signal at the annealing step of each PCR cycle was recorded for further analysis. The interpretation criteria for the TAM-mPCR assay are provided in S2 Table. The assay was optimized and validated for analytical performance, including detection limit and specificity, as described in the S1 Text. The validated TAM-mPCR assay was used to screen all clinical specimens in triplicate. A specimen was interpreted as positive when all three replicates showed amplification signals, and when the PCR assay controls were positive.

### Statistical analysis

Data were analyzed using IBM SPSS Statistics for Windows, version 29.0 (IBM Corp., Armonk, NY, USA), and GraphPad Prism, version 10.3.1 (GraphPad Software, San Diego, CA, USA). The diagnostic performance of TAM-mPCR was evaluated against the reference standard using sensitivity, specificity, positive predictive value (PPV), negative predictive value (NPV), and accuracy with exact two-sided 95% confidence intervals calculated by the Clopper–Pearson method. Agreement was assessed using Cohen’s kappa, and paired results were compared using McNemar’s test. Performance estimates were not calculated when fewer than five reference-positive specimens were available; these findings were reported descriptively. Cq values were compared between sites using the two-sided Mann–Whitney U test. Site-specific positivity rates were compared using Pearson’s chi-square or Fisher’s exact test, as appropriate. All tests were two-sided, with *P* < 0.05 considered statistically significant.

## Results

### Optimization and analytical performance of the TAM-mPCR assay

**Fig 2:**
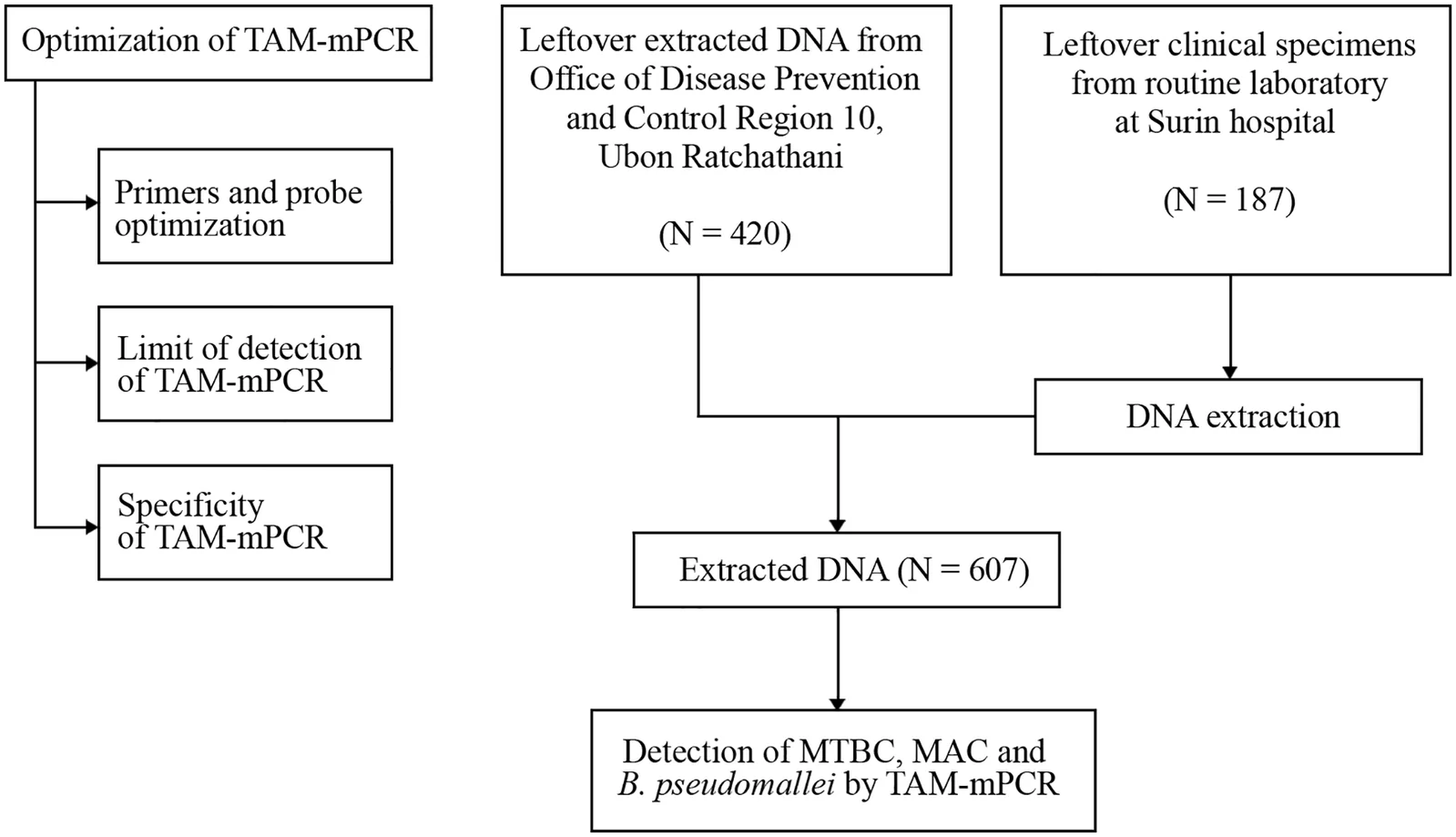
Flowchart of optimization and detection of MTBC, MAC, and *B. pseudomallei* in samples using TAM-mPCR assay.

The flowchart of TAM-mPCR assay optimization is shown in Fig 2. The primer concentrations at 0.4 µM for *IS6110*, *rrs*, and *BPSS1187*, and 0.8 µM for *RNase P*, provided optimal amplification (S1 Fig). A positive result was defined as Cq value ≤ 40 cycles.

The TAM-mPCR assay was capable of detecting the pathogen DNA, with LOD of 2 pg per reaction (≈ 4.2 × 10^2^ GE/reaction) for MTBC, 0.1 ng (≈ 1.9 × 10^4^ GE/reaction) for MAC, and 50 fg (≈ 6.3 GE/reaction) for *B. pseudomallei* (S2 Fig).

The TAM-mPCR assay showed 100% specificity, with no cross-reactivity observed when tested in triplicate against 16 non-target bacterial species, including *Acinetobacter baumannii*, *Acinetobacter lwoffii*, *Aeromonas hydrophila*, *Burkholderia cepacia*, *Burkholderia pickettii*, *Enterobacter aerogenes*, *Enterobacter cloacae*, *Enterococcus faecalis*, *Enterococcus faecium*, *Escherichia coli*, *Haemophilus influenzae*, *Klebsiella pneumoniae*, *Pseudomonas aeruginosa*, *Staphylococcus aureus*, *Streptococcus pneumoniae*, and *Streptococcus pyogenes* (S3 Fig).

### Diagnostic performance of the TAM-mPCR assay in pulmonary and extrapulmonary specimens

A total of 607 clinical specimens, comprising 504 pulmonary and 103 extrapulmonary specimens, were evaluated using TAM-mPCR for the detection of MTBC, MAC, *B. pseudomallei*, and co-infection (Table 2). Reference standards were defined separately for each target; therefore, the number of specimens included in each target-specific analysis varied according to the availability of an applicable reference result. All 79 specimens with other bacterial infections or no bacterial growth were negative for all TAM-mPCR targets (S3 Table).

**Table 2:** Overall and specimen group-stratified diagnostic performance of TAM-mPCR for the detection of MTBC, MAC, *B. pseudomallei*, and MTBC– *B. pseudomallei* co-infection.

Target organism	Specimen group	No. of samples	TP	FP	FN	TN	Sensitivity (%) (95% CI)	Specificity (%) (95% CI)	PPV (%) (95% CI)	NPV (%) (95% CI)	Accuracy (%) (95% CI)	Kappa (κ) (95% CI)
MTBC	Overall	521	330	2	0	189	100 (330/330) (98.9 – 100)	99.0 (189/191) (96.3 – 99.9)	99.4 (97.8 – 99.9)	100 (98.1 – 100)	99.6 (98.6 – 100)	0.99 (0.98 – 1.00)
	Pulmonary	449	321	2	0	126	100 (321/321) (98.9 – 100)	98.4 (126/128) (94.5 – 99.8)	99.4 (97.8 – 99.9)	100 (97.1 – 100)	99.6 (98.4 – 99.9)	0.99 (0.97 – 1.00)
	Extrapulmonary	72	9	0	0	63	100 (9/9) (66.4 – 100)	100 (63/63) (94.3 – 100)	100 (66.4 – 100)	100 (94.3 – 100)	100 (95.0 – 100)	1.00 (1.00 – 1.00)
MAC	Overall	104	3	0	0	101	NE	NE	NE	NE	NE	NE
	Pulmonary	91	2	0	0	89	NE	NE	NE	NE	NE	NE
	Extrapulmonary	13	1	0	0	12	NE	NE	NE	NE	NE	NE
*B. pseudomallei* ^a^	Overall	120	30	0	11^b^	79	73.2 (30/41) (57.1 – 85.8)	100 (79/79) (95.4 – 100)	100 (88.4 – 100)	87.8 (79.2 – 93.7)	90.8 (84.2 – 95.3)	0.78 (0.67 – 0.90)
	Pulmonary	86	18	0	10	58	64.3 (18/28) (44.1 – 81.4)	100 (58/58) (93.8 – 100)	100 (81.5 – 100)	85.3 (74.6 – 92.7)	88.4 (79.7 – 94.3)	0.71 (0.55 – 0.87)
	Extrapulmonary	34	12	0	1	21	92.3 (12/13) (64.0 – 99.8)	100 (21/21) (83.9 – 100)	100 (73.5 – 100)	95.5 (77.2 – 99.9)	97.1 (84.7 – 99.9)	0.94 (0.82 – 1.00)
Co-infection	Overall	36	2	0	0	34	NE	NE	NE	NE	NE	NE
	Pulmonary	33	2	0	0	31	NE	NE	NE	NE	NE	NE
	Extrapulmonary	3	0	0	0	3	NE	NE	NE	NE	NE	NE

TP, true positive; FP, false positive; FN, false negative; TN, true negative; PPV, positive predictive value; NPV**,** negative predictive value; CI, confidence interval; NE, not estimated. ^a^, All *B. pseudomallei* analyses, including the overall, pulmonary, and extrapulmonary categories, were based exclusively on specimens collected at Surin Hospital. ^b^, For *B. pseudomallei*, the 11 TAM-mPCR-negative specimens were obtained from patients in whom *B. pseudomallei* was cultured from different clinical specimens. These results therefore represent unmatched clinical-specimen comparisons rather than confirmed false-negative TAM-mPCR results. Diagnostic performance estimates were not calculated for MAC or co-infection because fewer than five reference-positive specimens were available. Exact two-sided 95% confidence intervals for sensitivity, specificity, PPV, NPV, and accuracy were calculated using the Clopper–Pearson method. The 95% confidence intervals for Cohen’s kappa coefficients were calculated using the asymptotic standard error.

In the overall MTBC analysis, TAM-mPCR achieved 100% sensitivity, 99.0% specificity, a PPV of 99.4%, an NPV of 100%, and near-perfect agreement with the reference standard (κ = 0.99) (Table 2). Sensitivity was 100% in both pulmonary (321/321) and extrapulmonary (9/9) specimens, with specificities of 98.4% and 100%, respectively.

All three culture-confirmed MAC specimens, including two pulmonary specimens and one extrapulmonary specimen, were detected by TAM-mPCR, with no positive results among 101 culture-negative specimens.

For *B. pseudomallei* detection, TAM-mPCR was positive in 30 of 41 AFB-negative specimens from patients with culture-confirmed melioidosis. Compared with culture, TAM-mPCR showed 73.2% sensitivity, 100% specificity, an NPV of 87.8%, and substantial agreement (κ = 0.78) (Table 2). However, in the TAM-mPCR negative cases, AFB-negative sputum or urine was tested, whereas *B. pseudomallei* was cultured from blood or pus from the same patients (S4 Table). All 11 TAM-mPCR-tested specimens showed valid *RNase P* amplification (Cq range, 21.2–35.3) but no *BPSS1187* amplification.

TAM-mPCR detected MTBC–*B. pseudomallei* co-infection in two specimens. Sequencing of the pathogen-specific amplicons confirmed both target identities (S4 Fig).

Site-stratified analysis showed 100% MTBC sensitivity at both sites, with specificities of 100% at Ubon and 95.6% at Surin. TAM-mPCR performance for *B. pseudomallei* was evaluated only at Surin (S5 Table). Median *RNase P* Cq values were lower at Surin than at Ubon in both overall and sputum analyses (*P* < 0.001) (S6 Table and S5 Fig). MTBC and *B. pseudomallei* positivity rates also differed between sites (S7 Table), whereas *IS6110* and *rrs* Cq values in culture-confirmed MTBC specimens were not significantly different (S8 Table).

### Comparison of TAM-mPCR assay results with AFB, culture, and NAAT methods

TAM-mPCR assay showed variable performance across target pathogens and reference methods (Table 3). For MTBC detection, TAM-mPCR showed 83.0% sensitivity, 95.9% specificity, and substantial agreement with AFB microscopy (κ = 0.72), with a statistically significant difference between the two methods (McNemar’s test, *P* < 0.001). In contrast, when compared with culture, the assay showed 100% sensitivity and 86.8% specificity, and substantial agreement (κ = 0.89), with no significant difference (*P* = 0.063). Compared with NAAT, the assay achieved 100% sensitivity and 99.5% specificity, with near-perfect agreement (κ = 0.996) and no significant difference (*P* = 1.000).

**Table 3:** Comparative diagnostic performance of the TAM-mPCR assay against AFB, culture, and NAAT for detection of MTBC, MAC, *B. pseudomallei*, and MTBC-*B. pseudomallei* co-infection.

Target organism	Method comparison	No. of sample	Sensitivity (%) (95% CI)	Specificity (%) (95% CI)	Kappa (κ) (95% CI)	McNemar *P*–value
MTBC	TAM-mPCR vs AFB	488	83 (284/342) (78.6 – 86.9)	95.9 (140/146) (91.3 – 98.5)	0.72 (0.65 – 0.78)	< 0.001
	TAM-mPCR vs Culture	104	100 (66/66) (94.6 – 100)	86.8 (33/38) (71.9 – 95.6)	0.89 (0.80 – 0.98)	0.063
	TAM-mPCR vs NAAT	501	100 (318/318) (98.9 – 100)	99.5 (182/183) (97.0 – 100)	0.996 (0.99 – 1.00)	1.000
MAC	TAM-mPCR vs AFB	488	0.6 (2/342) (0.1 – 2.1)	100 (146/146) (97.5 – 100)	0.004 (-0.001 – 0.008)	< 0.001
	TAM-mPCR vs Culture	104	3/3^a^	101/101^a^	NE	NE
	TAM-mPCR vs NAAT	501	NA^b^	498/501^b^	NE	NE
*B. pseudomallei* ^c^	TAM-mPCR vs Culture	120	73.2 (30/41) (57.1 – 85.8)	100 (79/79) (95.4 – 100)	0.78 (0.66 – 0.90)	0.001
Co-infection	TAM-mPCR vs Culture and/or Sequencing	36	2/2^a^	34/34^a^	NE	NE

AFB, acid-fast bacilli; NAAT, nucleic acid amplification test; CI, confidence interval; NA, not applicable; NE, not estimated or not performed, as appropriate. ^a^, Percentages, confidence intervals, kappa coefficients, and McNemar *P*–values were not estimated because fewer than five comparator-positive specimens were available; results are presented as n/N. ^b^, The available NAAT did not provide MAC-specific identification. Therefore, MAC-positive agreement could not be determined, and the reported negative proportion should not be interpreted as diagnostic specificity for MAC. ^c^, For *B. pseudomallei*, the 11 TAM-mPCR-negative specimens were obtained from patients in whom *B. pseudomallei* was cultured from different clinical specimens. These results therefore represent unmatched clinical-specimen comparisons rather than confirmed false-negative TAM-mPCR results. Exact two-sided 95% confidence intervals for proportions were calculated using the Clopper–Pearson method. The 95% confidence intervals for Cohen’s kappa coefficients were calculated using the asymptotic standard error.

For MAC detection, the TAM-mPCR assay showed poor agreement when compared with AFB microscopy (κ = 0.004), with a significant difference between methods (*P* < 0.001). TAM-mPCR detected all three culture-confirmed MAC specimens, while all 101 culture-negative specimens were TAM-mPCR-negative. Among the 501 specimens evaluated by NAAT, 498 were MAC-negative by TAM-mPCR.

For *B. pseudomallei* detection, TAM-mPCR showed 73.2% sensitivity, 100% specificity, and substantial agreement with culture (κ = 0.78), despite a significant difference between the methods (McNemar’s test, *P* = 0.001). TAM-mPCR correctly identified both MTBC–*B. pseudomallei* co-infections, and all 34 reference-negative specimens.

### Distribution and incremental value of TAM-mPCR over routine methods

The distribution and incremental yield of TAM-mPCR results over routine methods are shown in Table 4. Among AFB-positive specimens, MTBC and MAC were detected by TAM-mPCR in 82.7% (283/342) and 0.6% (2/342), respectively. One MTBC–*B. pseudomallei* co-infection (0.3%, 1/342) was identified. Interestingly, *B. pseudomallei* was detected in 20.5% (30/146) among AFB-negative specimens (n = 146), whereas MTBC was identified in 4.1% (6/146).

**Table 4:** Comparative TAM-mPCR detection results stratified by AFB, culture and NAAT methods.

Assay/result interpretation	Number of samples detected by TAM-mPCR (%)					
	Total samples	MTBC	MAC	*B. pseudomallei*	MTBC and *B. pseudomallei*	No target
**AFB (N = 488)**						
Positive	342 (70.1)	283 (82.7)	2 (0.6)	0	1 (0.3)	56 (16.4)
Negative	146 (29.9)	6 (4.1)	0	30 (20.5)	0	110 (75.3)
**Culture (N = 145)**						
MTBC	66 (45.5)	65 (98.5)	0	0	1 (1.5)	0
MAC	3 (2.1)	0	3 (100)	0	0	0
*M. scrofulaceum*	1 (0.7)	0	0	0	0	1 (100)
*B. pseudomallei*^a^	41 (28.3)	0	0	30 (73.2)	0	11 (26.8)
Negative	34 (23.4)	5 (14.7)	0	0	0	29 (85.3)
**NAAT (N = 501)**						
MTBC detected	318 (63.5)	316 (99.4)	0	0	2 (0.6)	0
NTM^b^ detected	7 (1.4)	0	0	0	0	7 (100)
No target detected	176 (35.1)	1 (0.6)	3 (1.7)	6 (3.4)	0	166 (94.3)

AFB, acid-fast bacilli; NAAT, nucleic acid amplification test; NTM, nontuberculous mycobacteria. ^a^, For *B. pseudomallei*, the 11 TAM-mPCR-negative specimens were obtained from patients in whom *B. pseudomallei* was cultured from different clinical specimens. These results therefore represent unmatched clinical-specimen comparisons rather than confirmed false-negative TAM-mPCR results. ^b^, NTM detected by NAATs were not specify NTM species.

For culture-confirmed MTBC cases, TAM-mPCR detected MTBC in 98.5% (65/66), with one MTBC–*B. pseudomallei* co-infection (1.5%, 1/66). All culture-confirmed MAC specimens were correctly detected by TAM-mPCR (100%, 3/3). Among culture-confirmed *B. pseudomallei* cases, TAM-mPCR detected *B. pseudomallei* in 73.2% (30/41). TAM-mPCR provided additional diagnostic cases among culture-negative specimens, detecting MTBC in 14.7% (5/34).

Among NAAT-positive MTBC specimens, TAM-mPCR detected MTBC in 99.4% (316/318), with two MTBC–*B. pseudomallei* co-infections (0.6%, 2/318). Among NAAT-negative specimens, TAM-mPCR detected additional cases, including *B. pseudomallei* in 3.4% (6/176), MAC in 1.7% (3/176), and MTBC in 0.6% (1/176). All NTM-detected specimens without species level by NAAT yielded no detectable targets by TAM-mPCR.

## Discussion

Accurate diagnosis of TB, NTM disease, and melioidosis remains challenging because of overlapping clinical presentations and limitations of conventional diagnostic methods. We developed a TAM-mPCR assay for the simultaneous detection of MTBC, MAC, and *B. pseudomallei*. The assay demonstrated strong diagnostic performance for MTBC, MAC, showed potential for *B. pseudomallei* detection, and identified MTBC–*B. pseudomallei* co-infections.

For MTBC, TAM-mPCR showed 100% sensitivity, 99.0% specificity, and an NPV of 100%, indicating its rule-out value in this study population. Sensitivity was 100% for both pulmonary and extrapulmonary specimens, with specificity of 98.4% and 100%, respectively. Agreement with NAAT was particularly high, with 100% sensitivity and 99.5% specificity. AFB microscopy produced more positive results than TAM-mPCR because it cannot distinguish MTBC from NTM, including MAC [44,45]. This limitation may lead to unnecessary anti-tuberculosis treatment, emphasizing the need for confirmation by molecular testing or mycobacterial culture [46].

Compared with mycobacterial culture, TAM-mPCR detected all 66 culture-positive specimens and five of 38 culture-negative specimens, resulting in a specificity of 86.8%. These discordant results may reflect limitations of culture, including low organism burden, poor specimen quality, prior antimicrobial exposure, contamination, or loss of organism viability during processing [47–52]. Conversely, molecular assays may detect DNA from nonviable organisms and therefore may not indicate active disease, while their performance can be affected by low bacterial burden, PCR inhibitors, poor specimen quality, and limited laboratory infrastructure [53]. Accordingly, TAM-mPCR results should be interpreted together with clinical, radiological, and other microbiological findings rather than used as a stand-alone indicator of active tuberculosis.

For MAC, TAM-mPCR detected all three culture-confirmed specimens, including two pulmonary and one extrapulmonary specimen, with no positive results among 101 culture-negative specimens. However, these findings should be interpreted cautiously and considered preliminary because of the small number of MAC-positive specimens. The ability to differentiate MAC from MTBC may be clinically valuable because AFB microscopy lacks species-level resolution [44,45], whereas culture-based identification is time-consuming [47–52].

For *B. pseudomallei*, TAM-mPCR showed 73.2% sensitivity, 100% specificity, and an NPV of 87.8%. The lower sensitivity and NPV in pulmonary specimens (64.3% and 85.3%, respectively) should be interpreted in the context of the study design. In 11 TAM-mPCR-negative cases, sputum or urine was tested, whereas culture detected *B. pseudomallei* in blood or pus from the same patients. These unmatched comparisons should not be considered confirmed false negatives or evidence of lower pulmonary performance. TAM-mPCR detected all 30 cases evaluated using matched specimens (30/30), supporting its potential utility. Therefore, unlike its strong rule-out performance for MTBC, a negative TAM-mPCR result from a residual AFB-tested specimen cannot exclude melioidosis at another anatomical site.

Across the two study sites, MTBC sensitivity was 100%, with specificities of 100% at Ubon and 95.6% at Surin. Although *RNase P* Cq values and target positivity rates differed between sites, *IS6110* and *rrs* Cq values among culture-confirmed MTBC specimens were comparable. Because the processing protocols were not applied in parallel to the same specimens, these differences cannot be attributed directly to sputum processing or DNA extraction, although NALC–NaOH processing and sputum DNA-extraction methods may affect mycobacterial recovery and molecular detection [54,55]. Differences in sampling periods, patient populations, specimen selection, and reference-testing availability may also have contributed; therefore, direct comparisons between sites should be interpreted cautiously.

Although MTBC–*B. pseudomallei* co-infection is considered uncommon, increasing reports suggest that it may be under-recognized in endemic regions [56]. Rubel et al. [27] described 14 TB–melioidosis co-infection cases reported over a decade in Brunei Darussalam and other parts of the WHO Western Pacific Region. Overlapping pulmonary and systemic manifestations may lead to diagnostic delay or misclassification. The immune-modulating effects of tuberculosis are well documented and are often characterized by chronic granulomatous inflammation that suppresses effective pathogen clearance. This may exacerbate the pathogenicity of *B. pseudomallei*, which thrives in a compromised immune microenvironment [56–58]. Additionally, tuberculosis-induced immunosuppression, particularly in the context of latent infections, may be activated or worsened by melioidosis [56,58,59]. For example, shared reliance on IFN-γ–mediated immunity may result in competitive immune resource depletion [6,56,60]. Moreover, overlapping treatments may cause adverse drug reactions and drug–drug interactions that compromise adherence and treatment effectiveness, although these effects require further investigation [56]. In this study, TAM-mPCR detected both MTBC and *B. pseudomallei* in two specimens for which reference testing did not include *B. pseudomallei* culture, and sequencing confirmed the identity of both targets.

The incremental detection achieved by TAM-mPCR highlights its value beyond routine diagnostic methods. Among AFB-negative specimens, *B. pseudomallei* and MTBC were detected only by TAM-mPCR in 20.5% and 4.1% of specimens, respectively, indicating that reliance on AFB microscopy alone may result in missed diagnoses. TAM-mPCR also detected additional MTBC among culture-negative specimens and multiple pathogens among NAAT-negative specimens. Although TAM-mPCR showed high concordance with NAAT for MTBC, it additionally enabled MAC identification and *B. pseudomallei* detection, which are not routinely provided by TB-focused assays. TAM-mPCR may be particularly valuable in regional laboratories where access to multiple molecular platforms is limited and culture turnaround times are prolonged [61–64]. Overall, its simultaneous detection capability may reduce underdiagnosis and improve disease surveillance by reducing the misclassification of melioidosis and NTM disease as tuberculosis [27,65–68], while facilitating recognition of co-infections that could be missed by conventional single-pathogen diagnostic workflows and supporting appropriate clinical management [27,56,69].

Several limitations of this study should be acknowledged. Only three MAC-positive specimens and two sequencing-confirmed co-infections were identified, precluding reliable estimation of diagnostic performance. The use of residual specimens without prospective enrollment or standardized inclusion criteria may limit generalizability. Sampling periods, sputum-processing procedures, and DNA-extraction methods also differed between sites and were not compared using the same specimens, which may have affected pathogen recovery, inhibitor removal, and performance estimates. For *B. pseudomallei*, 11 comparisons involved different specimens for TAM-mPCR and culture, preventing a matched assessment. In addition, because NPV depends on disease prevalence, the estimates obtained in this study may not be generalizable to populations with different prevalence. Future prospective studies are needed to validate the diagnostic performance of TAM-mPCR in larger and more diverse cohorts, including additional culture-confirmed MAC and other NTM specimens and co-infection cases. These studies should also evaluate TAM-mPCR using matched specimens, standardized procedures, and consistently applied target-specific reference standards.

In conclusion, TAM-mPCR provides a robust multiplex diagnostic approach for the simultaneous detection of MTBC, MAC, and *B. pseudomallei*. Its ability to identify alternative etiologies and MTBC–*B. pseudomallei* co-infections offers an important advantage over conventional single-target methods and may improve diagnostic yield, supporting its use as an adjunct tool in tuberculosis- and melioidosis-endemic settings. Further prospective studies are needed to define its clinical utility in routine practice.

## Supporting information

S1 Text Detailed methods for TAM-mPCR assay optimization [70–73]. (DOCX)

S1 Table Oligonucleotide primers and probes used in the TAM-mPCR assay for detection of *Mycobacterium tuberculosis* complex (MTBC), *Mycobacterium avium* complex (MAC) and *B. pseudomallei.* Primer and probe sequences are shown in the (5′ → 3′) orientation. Fluorophores and quenchers are indicated for probes. The *RNase P* target serves as an internal control for specimen and amplification validity.
(DOCX)

S2 Table Interpretation for TAM-mPCR detection of *Mycobacterium tuberculosis* complex (MTBC), *Mycobacterium avium* complex (MAC) and co-infections in clinical specimens. The assay included an internal control (*RNase P*) labeled with HEX, and three pathogen-associated targets: *IS6110* for MTBC (Texas-red), *rrs* for mycobacterial detection (Cy5), and *BPSS1187* for *B. pseudomallei* (FAM). The *rrs* is present in both MTBC and MAC; therefore, differentiation between MTBC and MAC relied on *IS6110*.
(DOCX)

S3 Table Results of the TAM-mPCR assay in sputum and non-sputum specimens with other bacterial infections (N = 49) or no bacterial growth (N = 30). (DOCX)

S4 Table Characteristics of unmatched TAM-mPCR-negative specimens obtained from patients with culture-confirmed *B. pseudomallei* infection. (DOCX)

S5 Table Site-stratified diagnostic performance of TAM-mPCR for the detection of MTBC, MAC, *B. pseudomallei*, and MTBC–*B. pseudomallei* co-infection. (DOCX)

S6 Table Comparison of RNase P Cq values between study sites. (DOCX)

S7 Table TAM-mPCR positivity rates for each target according to study site. (DOCX)

S8 Table Comparison of pathogen-specific TAM-mPCR Cq values among culture-confirmed positive specimens between study sites. (DOCX)

S1 Fig Optimization of primer concentration for *IS6110* (Texas Red), *rrs* (Cy5), *BPSS1187* (FAM) and *RNase P* (HEX). (A) Singleplex amplification of *IS6110* and *rrs* at primer concentrations ranging from 0.4 –1.0 µM. (B) Singleplex amplification of *BPSS1187* and *RNase P* at primer concentrations ranging from 0.4 –1.0 µM. (C) Multiplex amplification showing detection of MTBC using *IS6110* (Texas Red) and MAC using *rrs* (Cy5) at primer concentrations 0.4 µM. As the *rrs* target is present in both MTBC and MAC, differentiation between MTBC and MAC was based on the presence or absence of *IS6110*. (D) Multiplex amplification of *BPSS1187* (FAM) at primer concentrations 0.4 µM and *RNase P* (HEX) at primer concentrations 0.8 µM. Red circles indicate the selected primer concentrations. A primer concentration of 0.4 µM was required for amplification of *IS6110*, *rrs*, and *BPSS1187*, whereas a higher concentration (0.8 µM) was required for *RNase P*.
(DOCX)

S2 Fig Standard curves of TAM-mPCR were performed with 10-fold diluted DNA samples from *M. tuberculosis* 4871, *M. avium* subsp. *avium* 4968, *B. pseudomallei* K96243 and internal control. Fluorescence signals were obtained from Texas Red, Cy5, FAM and HEX dyes corresponding to the *IS6110*, *rrs*, *BPSS1187* and *RNase P* targets, respectively. (A) Amplification curves for *IS6110* targeting MTBC. (B) Amplification curves for *rrs* targeting MAC. (C) Amplification curves for *BPSS1187* targeting *B. pseudomallei*. (D) Amplification curves for *RNase P* as an internal control. Red boxes indicate the lowest DNA concentration consistently producing amplification (limit of detection, LOD) for each target. A positive result was defined as amplification with a Cq value ≤ 40 cycles.
(DOCX)

S3 Fig Specificity of TAM-mPCR. Fluorescence signals were obtained from Texas Red, Cy5, FAM and HEX dyes corresponding to the *IS6110*, *rrs*, *BPSS1187* and *RNase P* targets, respectively. TAM-mPCR assay was performed with DNA from various bacterial pathogens including positive and negative controls. (A) Specificity of the TAM-mPCR assay against non-target bacterial pathogens. A positive result was defined as amplification with a Cq value ≤ 40 cycles. Cq values > 40 were considered negative or non-specific background signals. (B) Amplification curves for *IS6110* showing specific detection of MTBC using positive control *M. tuberculosis* DNA, with no amplification observed advances to non-target bacterial DNA. (C) Amplification curves for *rrs* demonstrating specific amplification in MAC-positive samples, while no amplification was detected in non-mycobacterial controls. (D) Amplification curves for *BPSS1187* and *RNase P* showing specific detection of *B. pseudomallei* K96243, together with *RNase P* (HEX) amplification as an internal control.
(DOCX)

S4 Fig BLASTn analysis of Sanger-sequenced amplicons from two clinical specimens with MTBC–*B. pseudomallei* co-detection. Two target-specific amplicons from each clinical specimen were subjected to Sanger sequencing. The BLASTn results for samples 1 and 2 are presented in panels A and B, respectively. The resulting sequences were analyzed using NCBI BLASTn against the nucleotide collection (nt) database.
(DOCX)

S5 Fig Boxplot of RNase P Cq values by study site. (A) The boxplots show the distribution of *RNase P* Cq values in all clinical specimens collected from the Ubon Ratchathani Center and Surin Hospital. (B) The boxplots show the distribution of *RNase P* Cq values in sputum specimens collected from the Ubon Ratchathani Center and Surin Hospital. The central line represents the median, the box indicates the interquartile range (IQR), whiskers represent 1.5 × IQR, and circles indicate outliers. *P*-values were calculated using the Mann–Whitney U test.
(DOCX)

S1 Data Raw dataset for the evaluation of the multiplex PCR (TAM-mPCR) assay. (XLSX)
