# Supplementary material for: Development and evaluation of a multiplex PCR for simultaneous detection of Mycobacterium tuberculosis complex, Mycobacterium avium complex, and Burkholderia pseudomallei in clinical specimens
Source: PLoS Negl Trop Dis. 2026 Sep 24;20(9):e0014730. doi: 10.1371/journal.pntd.0014730 (PMC13606991; doi:10.1371/journal.pntd.0014730)
Supplement: Supplementary file 1 [file pntd.0014730.s001.docx]

**S1 Text**

**Methods and Results**

**Methods**

**Optimization of TAM-mPCR assay**

An in-house quadruplex real-time PCR assay, termed TAM-mPCR was developed using primers and probes targeting *IS6110* MTBC-specific insertion sequence, the conserved 16S ribosomal RNA region of MTBC and MAC (*rrs*), *BPSS1187* of *B. pseudomallei* [41-42], and the human *RNase P* gene as DNA extraction and PCR assay controls [43] (**S1 Table**).

The TAM-mPCR assay was performed in a total volume of 20 µL containing a 4× Multiplex PCR Master Mix (QuantiNova Multiplex PCR Kit, Qiagen, Australia), 0.8 µM *RNase P* primers, 0.4 µM *IS6110* primer, 0.4 µM *rrs* primer and 0.4 µM *BPSS1187* primer, 0.25 µM of each TaqMan probe and 2.5 µL template DNA. The reaction was performed using CFX96 Touch Real-Time PCR System (Bio-Rad, Hercules, CA, USA) with an initial denaturation at 95°C for 2 min, followed by 45 cycles of denaturation at 95°C for 5 seconds and combined annealing/extension step at 60°C for 30 seconds. Positive and negative (no-template) controls were included in each screening. Each fluorescence signal at the annealing step of each PCR cycle was recorded for further analysis. The interpretation criteria for the TAM-mPCR assay are provided in **S2 Table**.

To optimize the TAM-mPCR assay, the primer concentration was varied (1, 0.8, 0.75, 0.6, 0.45, or 0.4 µM), while the probe concentration was fixed at 0.25 µM (**S1 Fig**). The optimized assay was then validated for both detection limit (**S2 Fig**) and specificity for each target (**S3 Fig**). The limit of detection (LOD) of the TAM-mPCR assay was determined using tenfold serial dilution of target gDNA. Genome equivalents (GE) per reaction was calculated by dividing the DNA mass added to each reaction, expressed in femtograms, by the estimated mass of one genome [70]: **GE/reaction = DNA mass per reaction (fg)/estimated genome mass (fg/GE)**. GE was estimated based on a given genome weight [71]: 1 GE of MTBC 4871 equals 4.8 fg [72], 5.4 fg of MAC 4968 [73], and B. pseudomallei K96243 equals 7.9 fg [71]. DNA mass was converted using 1 pg = 10^3^ fg and 1 ng = 10^6^ fg. The tested dilution ranges were approximately 4.2×10^6^ to 0.004 GE/reaction for MTBC, 1.9×10^6^ to 0.002 GE/reaction for MAC, and 6.3×10^6^ to 0.006 GE/reaction for *B. pseudomallei*. Each concentration was tested in triplicate, and the lowest concentration detected in all three replicates was defined as the LOD. Assay specificity was evaluated in triplicate using 10 ng of DNA from each of 15 non-target bacterial strains (**S3 Fig**).

The validated TAM-mPCR assay was used to screen all clinical specimens in triplicate. A specimen was interpreted as positive when all three replicates showed amplification signals, and when the PCR assay controls for each replicate were positive.

**Preparation of positive DNA controls**

Genomic DNA from the MTBC 4871 and MAC 4968 (clinical strains) was used as positive control. These strains were previously identified as *M. tuberculosis* and *M. avium* using the Anyplex MTB/NTM Real-Time Detection kit (Seegene Inc., Seoul, Republic of Korea) and culture-based methods. Genomic DNA from *B. pseudomallei* strain K96243 was used as a positive control for *B. pseudomallei*. Human genomic DNA, obtained from our previous study [41], was used as an internal control. DNA quality was assessed by electrophoresis on a 0.8% agarose gel, and DNA purity and concentration were determined by measuring absorbance at 260 nm and 280 nm using a NanoDrop instrument (Thermo Scientific, USA). All DNA extracts used as positive and internal controls were confirmed by singleplex PCR targeting the *IS6110*, *rrs*, *BPSS1187* and *RNase P* genes. The resulting amplicons were verified by Sanger sequencing.

**Results**

**Optimization of TAM assay**

Optimization of primer concentrations demonstrated that 0.4 µM provided optimal amplification efficiency and consistency for *IS6110*, *rrs*, and *BPSS1187*, while *RNase P* required a higher concentration of 0.8 µM to achieve consistent internal control amplification across all tested conditions (**S1 Fig**).

Analytical sensitivity testing using ten-fold serial dilutions of reference genomic DNA showed that the TAM-mPCR assay was capable of detecting target DNA with LOD at 2 pg per reaction for MTBC (approximately 4.2 × 10^2^ GE/reaction), 0.1 ng per reaction for MAC (approximately 1.9 × 10^4^ GE/reaction), and 50 fg per reaction for *B. pseudomallei* (approximately 6.3 GE/reaction). Consistent amplification was observed at the LOD concentrations in all replicates, confirming reproducibility of the assay at the defined LOD (**S2 Fig**).

Specificity evaluation using DNA from 15 non-target bacterial species demonstrated no detectable cross-reactivity. All non-target organisms yielded negative amplification signals across triplicate testing, while positive controls consistently produced expected amplification curves (**S3 Fig**). These results support the high sensitivity and specificity of the assay for simultaneous detection of MTBC, MAC, and *B. pseudomallei* in clinical specimens.
