# Supplementary material for: Development and evaluation of a multiplex PCR for simultaneous detection of Mycobacterium tuberculosis complex, Mycobacterium avium complex, and Burkholderia pseudomallei in clinical specimens
Source: PLoS Negl Trop Dis. 2026 Sep 24;20(9):e0014730. doi: 10.1371/journal.pntd.0014730 (PMC13606991; doi:10.1371/journal.pntd.0014730)
Supplement: Supplementary file 2 [file pntd.0014730.s002.docx]

**S1 Table.** Oligonucleotide primers and probes used in the TAM-mPCR assay for detection of *Mycobacterium tuberculosis* complex (MTBC), *Mycobacterium avium* complex (MAC) and *B. pseudomallei*. Primer and probe sequences are shown in the (5′→3′) orientation. Fluorophores and quenchers are indicated for probes. The *RNase P* target serves as an internal control for specimen and amplification validity.

| Target genes | Primers/Probes | Sequences (5′→3′) | Target organisms | References |
| --- | --- | --- | --- | --- |
| *IS6110*  (Insertion sequence) | *IS6110*-F | CGAACTCAAGGAGCACATCAG | MTBC | J. Thaipadung  panit, unpublished data |
|  | *IS6110*-R | AGTTTGGTCATCAGCCGTTC |  |  |
|  | *IS6110*-P | TexasRed-AAAGTGTGGCTAACCCTGAACCGT-BHQ2 |  |  |
| 16S ribosomal RNA | *rrs*-F3 | CATGTCTTGTGGTGGAAAGC | Mycobacteria (MTBC and MAC) | J. Thaipadungpanit, unpublished data |
|  | *rrs*-R | CGTATCTCAGTCCCAGTGTG |  |  |
|  | *rrs*-P | Cy5-CGGTGTGGGATGAGCCCGC-BHQ2 |  |  |
| *BPSS1187*  (Hypothetical protein) | 8653-F | ATCGAATCAGGGCGTTCAAG | *B. pseudomallei* | 41-42 |
|  | 8653-R | CATTCGGTGACGACACGACC |  |  |
|  | 8653-P | FAM-CGCCGCAAGACGCCATCGTTCAT-TAMRA |  |  |
| *RNase P*  (Ubiquitous ribonucleoprotein complex) | *RNase P*-F | AGATTTGGACCTGCGAGCG | Human  (Internal control) | 43 |
|  | *RNase P*-R | GAGCGGCTGTCTCCACAAGT |  |  |
|  | *RNase P*-P | HEX-TTCTGACCTGAAGGCTCTGCGCG-BHQ1 |  |  |
