# Supplementary material for: Development and evaluation of a multiplex PCR for simultaneous detection of Mycobacterium tuberculosis complex, Mycobacterium avium complex, and Burkholderia pseudomallei in clinical specimens
Source: PLoS Negl Trop Dis. 2026 Sep 24;20(9):e0014730. doi: 10.1371/journal.pntd.0014730 (PMC13606991; doi:10.1371/journal.pntd.0014730)
Supplement: Supplementary file 3 [file pntd.0014730.s003.docx]

**S2 Table.** Interpretation for TAM-mPCR detection of *Mycobacterium tuberculosis* complex (MTBC), *Mycobacterium avium* complex (MAC) and co-infections in clinical specimens. The assay included an internal control (*RNase P*) labeled with HEX, and three pathogen-associated targets: *IS6110* for MTBC (Texas-red), *rrs* for mycobacterial detection (Cy5), and *BPSS1187* for *B. pseudomallei* (FAM). The *rrs* is present in both MTBC and MAC; therefore, differentiation between MTBC and MAC relied on *IS6110*.

| Interpretation | *IS6110*  (Texas-red) | | *rrs*  (Cy5) | *BPSS1187*  (FAM) | *RNase P*  (HEX) |
| --- | --- | --- | --- | --- | --- |
| MTBC detected | + | + | | - | + |
| MAC (*M. avium*) detected | - | + | | - | + |
| MTBC and B. pseudomallei detected | + | + | | + | + |
| MAC and B. pseudomallei detected | - | + | | + | + |
| B. pseudomallei detected | - | - | | + | + |
| Not detected (no target detected) | - | - | | - | + |
