# Supplementary material for: Development and evaluation of a multiplex PCR for simultaneous detection of Mycobacterium tuberculosis complex, Mycobacterium avium complex, and Burkholderia pseudomallei in clinical specimens
Source: PLoS Negl Trop Dis. 2026 Sep 24;20(9):e0014730. doi: 10.1371/journal.pntd.0014730 (PMC13606991; doi:10.1371/journal.pntd.0014730)
Supplement: Supplementary file 4 [file pntd.0014730.s004.docx]

**S3 Table**. Results of the TAM-mPCR assay in sputum and non-sputum specimens with other bacterial infections (N = 49) or no bacterial growth (N = 30).

| **Specimens** | **Culture result** | **No. of samples** | **TAM result** |
| --- | --- | --- | --- |
| Sputum | *Acinetobacter baumannii* | 10 | No target detected |
|  | *Acinetobacter lwoffii* | 1 | No target detected |
|  | *Acinetobacter nosocomialis* | 1 | No target detected |
|  | *Achromobacter xylosoxidans* | 1 | No target detected |
|  | *Aspergillus* sp. | 1 | No target detected |
|  | *Burkholderia cepacia* | 2 | No target detected |
|  | *Candida albicans* | 2 | No target detected |
|  | *Escherichia coli* | 2 | No target detected |
|  | *Klebsiella oxytoca* | 1 | No target detected |
|  | *Klebsiella pneumoniae* | 8 | No target detected |
|  | *Nocardia* spp. | 1 | No target detected |
|  | *Proteus mirabilis* | 3 | No target detected |
|  | *Pseudomonas aeruginosa* | 4 | No target detected |
|  | *Staphylococcus aureus* | 2 | No target detected |
|  | *Stenotrophomonas maltophilia* | 1 | No target detected |
|  | Normal flora | 3 | No target detected |
|  | **No bacterial growth** | **9** | No target detected |
| Non-sputum | *Cryptococcus neoformans* | 1 | No target detected |
|  | *Streptococcus mitis* | 1 | No target detected |
|  | *Staphylococcus haemolyticus* | 1 | No target detected |
|  | *Enterobacter agglomerans* | 1 | No target detected |
|  | *Micrococcus* sp. | 1 | No target detected |
|  | *Klebsiella pneumoniae* | 1 | No target detected |
|  | **No bacterial growth** | **21** | No target detected |
