# Supplementary material for: Development and evaluation of a multiplex PCR for simultaneous detection of Mycobacterium tuberculosis complex, Mycobacterium avium complex, and Burkholderia pseudomallei in clinical specimens
Source: PLoS Negl Trop Dis. 2026 Sep 24;20(9):e0014730. doi: 10.1371/journal.pntd.0014730 (PMC13606991; doi:10.1371/journal.pntd.0014730)
Supplement: Supplementary file 5 [file pntd.0014730.s005.docx]

**S4 Table**. Characteristics of unmatched TAM-mPCR-negative specimens obtained from patients with culture-confirmed B. pseudomallei infection.

| **Specimen ID** | **Study site** | **Specimen category** | **Specimen tested by TAM-mPCR** | **Culture-positive specimen** | **Specimen pairing** | **RNase P Cq** | **BPSS1187 result** |
| --- | --- | --- | --- | --- | --- | --- | --- |
| MP-567 | Surin | Pulmonary | Sputum | Blood | Different | 21.2 | ND |
| MP-568 | Surin | Pulmonary | Sputum | Pus | Different | 23.8 | ND |
| MP-569 | Surin | Pulmonary | Sputum | Blood | Different | 25.5 | ND |
| MP-571 | Surin | Extrapulmonary | Urine | Pus | Different | 35.3 | ND |
| MP-581 | Surin | Pulmonary | Sputum | Pus | Different | 27.4 | ND |
| MP-586 | Surin | Pulmonary | Sputum | Pus | Different | 28.9 | ND |
| MP-587 | Surin | Pulmonary | Sputum | Pus | Different | 28.5 | ND |
| MP-588 | Surin | Pulmonary | Sputum | Pus | Different | 27.3 | ND |
| MP-590 | Surin | Pulmonary | Sputum | Blood | Different | 27.2 | ND |
| MP-592 | Surin | Pulmonary | Sputum | Blood | Different | 26.3 | ND |
| MP-593 | Surin | Pulmonary | Sputum | Pus | Different | 25.2 | ND |

AFB, acid-fast bacilli; Cq, quantification cycle; ND, not detected. All specimens listed in this table were residual AFB smear-negative specimens originally submitted for routine tuberculosis testing and were not collected specifically for B. pseudomallei detection. “Different” indicates that the two tests were performed using different clinical specimens from the same patient. *RNase P* served as an internal control for specimen adequacy, DNA extraction, and PCR amplification but did not directly assess the recovery of B. pseudomallei DNA.
