# Supplementary material for: Development and evaluation of a multiplex PCR for simultaneous detection of Mycobacterium tuberculosis complex, Mycobacterium avium complex, and Burkholderia pseudomallei in clinical specimens
Source: PLoS Negl Trop Dis. 2026 Sep 24;20(9):e0014730. doi: 10.1371/journal.pntd.0014730 (PMC13606991; doi:10.1371/journal.pntd.0014730)
Supplement: Supplementary file 6 [file pntd.0014730.s006.docx]

**S5 Table.** Site-stratified diagnostic performance of TAM-mPCR for the detection of MTBC, MAC, *B. pseudomallei*, and MTBC–B. pseudomallei co-infection.

| **Target organism** | **Study site** | **No. of sample** | **TP** | **FP** | **FN** | **TN** | **Sensitivity %**  **(95% CI)** | **Specificity % (95% CI)** | **PPV %**  **(95% CI)** | **NPV %**  **(95% CI)** | **Accuracy % (95% CI)** | **Kappa (κ)**  **(95% CI)** |
| --- | --- | --- | --- | --- | --- | --- | --- | --- | --- | --- | --- | --- |
| MTBC | Ubon | 420 | 274 | 0 | 0 | 146 | 100 (274/274)  (98.7 – 100) | 100 (146/146)  (97.5 – 100) | 100  (98.7 – 100) | 100  (97.5 – 100) | 100  (99.1 – 100) | 1.00  (1.00 – 1.00) |
|  | Surin | 101 | 56 | 2 | 0 | 43 | 100 (56/56)  (93.6 – 100) | 95.6 (43/45)  (84.9 – 99.5) | 96.6  (88.1 – 99.6) | 100  (91.8 – 100) | 98  (93.0 – 99.8) | 0.96  (0.91 – 1.00) |
| MAC | Ubon | 34 | 1 | 0 | 0 | 33 | NE | NE | NE | NE | NE | NE |
|  | Surin | 70 | 2 | 0 | 0 | 68 | NE | NE | NE | NE | NE | NE |
| *B. pseudomallei* | Surin | 120 | 30 | 0 | 11^a^ | 79 | 73.2 (30/41)  (57.1 – 85.8) | 100 (79/79)  (95.4 – 100) | 100  (88.4 – 100) | 87.8  (79.2 – 93.7) | 90.8  (84.2 – 95.3) | 0.78  (0.67 – 0.90) |
| Co-infection | Ubon | 1 | 1 | 0 | 0 | 0 | NE | NE | NE | NE | NE | NE |
|  | Surin | 35 | 1 | 0 | 0 | 34 | NE | NE | NE | NE | NE | NE |

TP, true positive; FP, false positive; FN, false negative; TN, true negative; PPV, positive predictive value; **NPV, negative predictive value;** CI, confidence interval; NE, not estimated**.** ^a^, For B. pseudomallei, the 11 TAM-mPCR-negative specimens were obtained from patients in whom B. pseudomallei was cultured from different clinical specimens. These results therefore represent unmatched clinical-specimen comparisons rather than confirmed false-negative TAM-mPCR results. Diagnostic performance for B. pseudomallei could not be evaluated at Ubon Ratchathani Center because no specimens had an applicable bacterial culture reference result. Diagnostic performance estimates were not calculated for MAC or co-infection because fewer than five reference-positive specimens were available. Exact two-sided 95% confidence intervals for sensitivity, specificity, PPV, NPV, and accuracy were calculated using the Clopper–Pearson method. The 95% confidence intervals for Cohen’s kappa coefficients were calculated using the asymptotic standard error.
