# Supplementary material for: Development and evaluation of a multiplex PCR for simultaneous detection of Mycobacterium tuberculosis complex, Mycobacterium avium complex, and Burkholderia pseudomallei in clinical specimens
Source: PLoS Negl Trop Dis. 2026 Sep 24;20(9):e0014730. doi: 10.1371/journal.pntd.0014730 (PMC13606991; doi:10.1371/journal.pntd.0014730)
Supplement: Supplementary file 7 [file pntd.0014730.s007.docx]

**S6 Table**. Comparison of *RNase P* Cq values between study sites.

| **Specimen group** | **Ubon Ratchathani Center** | | | **Surin Hospital** | | | ***P***–**value** |
| --- | --- | --- | --- | --- | --- | --- | --- |
|  | **No. of sample** | **Median Cq (IQR)** | **Cq range** | **No. of sample** | **Median Cq (IQR)** | **Cq range** |  |
| All specimens | 420 | 27.8 (26.2–29.7) | 22.6–34.6 | 187 | 26.7 (24.8–28.4) | 20.4–35.3 | < 0.001 |
| Sputum specimens | 351 | 27.7 (26.1–29.7) | 22.6–34.6 | 147 | 26.3 (24.7–27.6) | 20.6–34.2 | < 0.001 |

Cq, quantification cycle; IQR, interquartile range; NALC, N-acetyl-L-cysteine. *RNase P* Cq values were compared between study sites using the two-sided Mann–Whitney U test. *RNase P* served as an internal control for specimen adequacy, DNA extraction, and PCR amplification but did not directly measure pathogen DNA recovery.
