# Supplementary material for: Development and evaluation of a multiplex PCR for simultaneous detection of Mycobacterium tuberculosis complex, Mycobacterium avium complex, and Burkholderia pseudomallei in clinical specimens
Source: PLoS Negl Trop Dis. 2026 Sep 24;20(9):e0014730. doi: 10.1371/journal.pntd.0014730 (PMC13606991; doi:10.1371/journal.pntd.0014730)
Supplement: Supplementary file 8 [file pntd.0014730.s008.docx]

**S7 Table**. TAM-mPCR positivity rates for each target according to study site.

| **TAM-mPCR result** | **Ubon Ratchathani**  **Positive/total (%)** | **Surin**  **Positive/total (%)** | ***P***–**value** |
| --- | --- | --- | --- |
| Any MTBC detection | 274/420 (65.2) | 58/187 (31) | < 0.001 |
| Any MAC detected | 1/420 (0.2) | 2/187 (1.1) | 0.226 |
| Any *B. pseudomallei* detected | 1/420 (0.2) | 31/187 (16.6) | < 0.001 |
| Co-infection detection | 1/420 (0.2) | 1/187 (0.5) | 0.522 |

Target-specific positivity rates were not mutually exclusive. Specimens with simultaneous detection of MTBC and B. pseudomallei were included in the positivity counts for both targets and were also reported separately as co-detections. *P*-values were calculated using Pearson’s chi-square test or Fisher’s exact test, as appropriate.
