# Supplementary material for: Development and evaluation of a multiplex PCR for simultaneous detection of Mycobacterium tuberculosis complex, Mycobacterium avium complex, and Burkholderia pseudomallei in clinical specimens
Source: PLoS Negl Trop Dis. 2026 Sep 24;20(9):e0014730. doi: 10.1371/journal.pntd.0014730 (PMC13606991; doi:10.1371/journal.pntd.0014730)
Supplement: Supplementary file 9 [file pntd.0014730.s009.docx]

**S8 Table**. Comparison of pathogen-specific TAM-mPCR Cq values among culture-confirmed positive specimens between study sites.

| **Target**  **organism** | **Target gene** | **Ubon Ratchathani**  **Median Cq (IQR)** | **Surin**  **Median Cq (IQR)** | ***P*** – **value** |
| --- | --- | --- | --- | --- |
| MTBC | *IS6110* | 28.5 (27.1 – 29.6) | 29.0 (27.5 – 30.4) | 0.390 |
| MTBC | *rrs* | 28.2 (24.7 – 32.2) | 29.1 (27.4 – 30.7) | 0.376 |
| MAC | *rrs* | 32.5 (n = 1) | 30.0 (29.5 – 30.5; n = 2) | NA |
| *B. pseudomallei* | *BPSS1187* | – | 31.0 (27.8 – 32.1) | NA |

NA, not applicable; Cq, quantification cycle; IQR, interquartile range. Data are presented as median Cq (IQR), except for groups with fewer than five specimens, for which individual Cq values are shown. Cq values were compared between study sites using the Mann–Whitney U test. Statistical comparisons were not performed when fewer than five eligible specimens were available in one or both study sites. The symbol “–” indicates that no eligible culture-confirmed positive specimen was available.
