# Supplementary material for: Development and evaluation of a multiplex PCR for simultaneous detection of Mycobacterium tuberculosis complex, Mycobacterium avium complex, and Burkholderia pseudomallei in clinical specimens
Source: PLoS Negl Trop Dis. 2026 Sep 24;20(9):e0014730. doi: 10.1371/journal.pntd.0014730 (PMC13606991; doi:10.1371/journal.pntd.0014730)
Supplement: Supplementary file 10 [file pntd.0014730.s010.docx]

A.

C.

D.

B.

0.4 µM

0.6 µM

0.75 µM

0.8 µM

1 µM

*BPSS1187* (FAM)

*IS6110* (Texas-red)

*rrs* (Cy5)

*BPSS1187* (FAM)

*RNase P* (HEX)

*IS6110* (Texas-red)

*rrs* (Cy5)

*BPSS1187* (FAM)

*RNase P* (HEX)

**S1 Fig. Optimization of primer concentration for** *IS6110* (Texas Red)**,** rrs (Cy5)**,** *BPSS1187* (FAM) and *RNase P* (HEX). (A) Singleplex amplification of *IS6110* and rrs at primer concentrations ranging from 0.4 –1.0 µM. (B) Singleplex amplification of *BPSS1187* and *RNase P* at primer concentrations ranging from 0.4 –1.0 µM. (C) Multiplex amplification showing detection of MTBC using *IS6110* (Texas Red) and MAC using rrs (Cy5) at primer concentrations 0.4 µM. As the rrs target is present in both MTBC and MAC, differentiation between MTBC and MAC was based on the presence or absence of *IS6110*. (D) Multiplex amplification of *BPSS1187* (FAM) at primer concentrations 0.4 µM and *RNase P* (HEX) at primer concentrations 0.8 µM. Red circles indicate the selected primer concentrations. A primer concentration of 0.4 µM was required for amplification of *IS6110*, rrs, and *BPSS1187*, whereas a higher concentration (0.8 µM) was required for *RNase P*.
