# Supplementary material for: Development and evaluation of a multiplex PCR for simultaneous detection of Mycobacterium tuberculosis complex, Mycobacterium avium complex, and Burkholderia pseudomallei in clinical specimens
Source: PLoS Negl Trop Dis. 2026 Sep 24;20(9):e0014730. doi: 10.1371/journal.pntd.0014730 (PMC13606991; doi:10.1371/journal.pntd.0014730)
Supplement: Supplementary file 11 [file pntd.0014730.s011.docx]

A.

C.

B.

D.

**S2 Fig.** Standard curves of TAM-mPCR were performed with 10-fold diluted DNA samples from M. tuberculosis 4871, M. avium subsp. avium 4968, B. pseudomallei K96243 and internal control***.*** Fluorescence signals were obtained from Texas Red, Cy5, FAM and HEX dyes corresponding to the ***IS6110*, *rrs*, *BPSS1187*** and ***RNase P*** targets, respectively. **(A)** Amplification curves for *IS6110* targeting MTBC. **(B)** Amplification curves for rrs targeting MAC. **(C)** Amplification curves for *BPSS1187* targeting B. pseudomallei. **(D)** Amplification curves for *RNase P* as an internal control. Red boxes indicate the lowest DNA concentration consistently producing amplification (limit of detection, LOD) for each target. A positive result was defined as amplification with a Cq value ≤ 40 cycles.
