# Supplementary material for: Development and evaluation of a multiplex PCR for simultaneous detection of Mycobacterium tuberculosis complex, Mycobacterium avium complex, and Burkholderia pseudomallei in clinical specimens
Source: PLoS Negl Trop Dis. 2026 Sep 24;20(9):e0014730. doi: 10.1371/journal.pntd.0014730 (PMC13606991; doi:10.1371/journal.pntd.0014730)
Supplement: Supplementary file 12 [file pntd.0014730.s012.docx]

A.

B.

C.

D.

**S3 Fig**. Specificity of TAM-mPCR. Fluorescence signals were obtained from Texas Red, Cy5, FAM and HEX dyes corresponding to the ***IS6110*, *rrs*, *BPSS1187* and *RNase P*** targets, respectively. TAM-mPCR assay was performed with DNA from various bacterial pathogens including positive and negative controls. (A) Specificity of the TAM-mPCR assay against non-target bacterial pathogens. A positive result was defined as amplification with a Cq value ≤ 40 cycles. Cq values > 40 were considered negative or non-specific background signals. **(B)** Amplification curves for *IS6110* showing specific detection of MTBC using positive control M. tuberculosis DNA, with no amplification observed advances to non-target bacterial DNA. **(C)** Amplification curves for rrs demonstrating specific amplification in MAC-positive samples, while no amplification was detected in non-mycobacterial controls. **(D)** Amplification curves for *BPSS1187* and *RNase P* showing specific detection of B. pseudomallei K96243, together with *RNase P* (HEX) amplification as an internal control.
