# Supplementary material for: Development and evaluation of a multiplex PCR for simultaneous detection of Mycobacterium tuberculosis complex, Mycobacterium avium complex, and Burkholderia pseudomallei in clinical specimens
Source: PLoS Negl Trop Dis. 2026 Sep 24;20(9):e0014730. doi: 10.1371/journal.pntd.0014730 (PMC13606991; doi:10.1371/journal.pntd.0014730)
Supplement: Supplementary file 13 [file pntd.0014730.s013.docx]

**A.**

**B.**

**S4 Fig. BLASTn analysis of Sanger-sequenced amplicons from two clinical specimens with MTBC–B. pseudomallei co-detection.** Two target-specific amplicons from each clinical specimen were subjected to Sanger sequencing. The BLASTn results for samples 1 and 2 are presented in panels A and B, respectively. The resulting sequences were analyzed using NCBI BLASTn against the nucleotide collection (nt) database.
