# Supplementary material for: Development and evaluation of a multiplex PCR for simultaneous detection of Mycobacterium tuberculosis complex, Mycobacterium avium complex, and Burkholderia pseudomallei in clinical specimens
Source: PLoS Negl Trop Dis. 2026 Sep 24;20(9):e0014730. doi: 10.1371/journal.pntd.0014730 (PMC13606991; doi:10.1371/journal.pntd.0014730)
Supplement: Supplementary file 14 [file pntd.0014730.s014.docx]

**A.**

**B.**

**S5 Fig. Boxplot of RNase P Cq values by study site.** (A) The boxplots show the distribution of *RNase P* Cq values in all clinical specimens collected from the Ubon Ratchathani Center and Surin Hospital. (B) The boxplots show the distribution of *RNase P* Cq values in sputum specimens collected from the Ubon Ratchathani Center and Surin Hospital. The central line represents the median, the box indicates the interquartile range (IQR), whiskers represent 1.5 × IQR, and circles indicate outliers. *P*-values were calculated using the Mann–Whitney U test.
